# Impact of Vitamin D Status on Pancreatic Cancer Risk and Outcomes

**DOI:** 10.3390/nu18050837

**Published:** 2026-03-05

**Authors:** Beata Jabłońska, Sławomir Mrowiec

**Affiliations:** Department of Digestive Tract Surgery, Medical University of Silesia, Medyków 14, 40-752 Katowice, Poland

**Keywords:** pancreatic cancer, pancreatic ductal adenocarcinoma, tumor microenvironment, vitamin D, calcitriol

## Abstract

Vitamin D (VD), a fat-soluble prohormone, exerts diverse effects on cellular proliferation, differentiation, and immune modulation, with accumulating evidence supporting its role in pancreatic ductal adenocarcinoma (PDAC) biology. Experimental studies demonstrate that VD and its analogs can inhibit PDAC cell growth and remodel the tumor microenvironment, potentially contributing to tumor suppression. Epidemiological data indicate that VD deficiency is prevalent among PDAC patients and is associated with increased inflammatory biomarkers and reduced overall survival, particularly in early-stage disease. However, meta-analyses reveal inconsistent associations between circulating 25-hydroxyvitamin D levels and PDAC incidence, while higher levels may be linked to improved survival but not reduced risk of disease onset. The clinical utility of VD supplementation for PDAC prevention or treatment remains uncertain, with ongoing debate regarding optimal dosing, timing, and patient selection. This narrative review synthesizes current evidence on the mechanistic, epidemiological, and clinical relevance of VD in PDAC. Particular emphasis is placed on existing knowledge gaps and the need for well-designed clinical trials to clarify the potential therapeutic and prognostic role of VD in pancreatic cancer.

## 1. Introduction

Pancreatic ductal adenocarcinoma (PDAC) is characterized by poor prognosis. The incidence of this condition has recently increased. Currently, it is the 14th most frequent carcinoma and the 7th highest cause of cancer-related mortality worldwide [1,2]. Despite of surgery and oncological treatment, the 5-year overall survival (OS) rate is only approximately 5% [3].

Vitamin D (VD) is a vitamin that is soluble in fat and it is a prohormone. It is involved in various physiological and pathological processes in the human body. Calcium homeostasis is the main function of VD, but it also plays a role in the inhibition of numerous pleiotropic processes in many human organs [1]. VD participates in various cellular reactions, such as cell differentiation, proliferation, and apoptosis. Therefore, low plasma levels of VD may be a cause of cancer development and progression [4,5].

There are inconsistent data in the literature concerning the association between VD and PDAC. According to numerous studies, increased VD consumption is associated with a lower cancer risk, including PDAC [6,7,8]. According to other studies, increased VD consumption is associated with an increased cancer risk [6,9]. In addition, VD deficiency is present in most PDAC patients [6,10]. Moreover, according to some authors, higher plasma VD concentrations are associated with better survival in PDAC patients [6].

The tumor microenvironment (TME) in PDAC involves various pro- and anti-tumor components. Dense desmoplasia involves immune cells, the extracellular matrix, growth factors, cytokines, cancer-associated fibroblasts (CAFs), and pancreatic stellate cells (PSCs). There are many targets in PDAC, including hyaluronan, angiogenesis, focal adhesion kinase (FAK), connective tissue growth factor (CTGF), CD40, chemokine (C-X-C motif) receptor 4 (CXCR-4), immunotherapy, and VD. CAFs express α-smooth muscle actin (α-SMA). These factors significantly influence tumor progression. CAFs affect cancer growth, survival, metastasis, angiogenesis, and immunosurveillance through the secretion of various cytokines. Therefore, targeting CAFs can be used to treat patients with PDAC. Activation of the VD receptor (VDR) decreases fibrosis and pro-inflammatory markers and increases intra-tumoral gemcitabine levels in PDAC [11,12].

It has been reported that higher concentrations of serum 25-hydroxyvitamin D [25(OH)D] are associated with better survival in patients with PDAC [13]. Experimental studies have shown the anti-cancer effects of VD, including inhibition of cancer cell proliferation, induction of apoptosis and differentiation, and potentiation of chemotherapy or radiotherapy in various cancers [6,11]. Additionally, experimental studies have shown that VD regulates the tumor microenvironment, particularly CAF reprogramming, which facilitates tumor repression [6]. VDR signaling influences CAFs. VDR signaling inhibits CAF release from exosomal miR-10a-5p and limits its supportive effects on PDAC cells [14]. Dysregulated signaling of nuclear transcription factors VDR and Forkhead box M1 (FOXM1) is associated with cancer transformation and tumorigenesis in PDAC [15].

The goal of this study was to review the global literature concerning the association between VD and PDAC risk and survival.

## 2. Vitamin D Effect on the Immune System in PDAC

Tumor-associated macrophages (TAMs) are present in the tumor microenvironment (TME) of PDAC. TAMs play an important role in PDAC progression via the production of interleukin (IL)-6 and activation of the STAT3 pathway. They release IL-6 and activate the STAT3/SOCS pathway, which accelerates PDAC generation [12].

Polymorphonuclear neutrophil (PMN) infiltrates can be observed near tumor cells or in the stroma and are associated with poor differentiation. Neutrophils in PDAC are located within solid tumors as anti-tumor N1 TANs (Tumor-Associated Neutrophils) or pro-tumor N2 TANs [12]. CD4+ T lymphocytes play a role in promoting PanIN formation by intercepting anti-tumor immune responses by CD8+ T lymphocytes [12]. CD4+ T lymphocytes are present in the PDAC TME of Kras-dependent cancers. CD4+ T lymphocytes and IL-17 are required for oncogenic KRAS-driven PDAC. CD4+ T lymphocytes suppress the anti-tumor activity of CD8+ T lymphocytes by secreting IL-17. In addition, TH17 lymphocytes secrete IL-17A, indicating acinar–ductal metaplasia, and PanINs through IL-17RA, which induces tumor initiation and progression [12].

The significant role of VD in both the innate and adaptive immune systems is associated with the expression of VDR in almost all cells of the immune system, as well as the presence of metabolizing hormones in immune cells. In vitro studies have shown a clear impact of supraphysiological VD doses on the immune system [16,17]. VD affects the innate and adaptive immune systems, including monocytes/macrophages, and VD via VDR modulates the inflammatory response of dendritic cells, as well as natural killer (NK) cells, neutrophils, T-lymphocytes, and B-lymphocytes. This impact involves regulation of cytokine secretion [16,17,18].

## 3. TME in PDAC

The TME consists of various stromal cells (e.g., endothelia, CAFs, and immune cells) as well as extracellular components (e.g., cytokines and the extracellular matrix), which surround tumor cells and are nourished by the vascular system [19]. Pancreatic stellate cells (PSCs), which are star-shaped cells, are found in the normal exocrine pancreas. These cells are activated during neoplastic progression, acquire a myofibroblast-like phenotype, express α-SMA, and secrete extracellular matrix (ECM) proteins (collagen I, collagen III, collagen IV, and fibronectin). These activated PSCs are termed CAFs. The ECM proteins secreted/deposited by CAFs, with high-molecular-weight glycosaminoglycans such as hyaluronan (HA), lead to an increase in the interstitial pressure, causing vascular collapse, which leads to the formation of a hypoxic and nutrient-poor TME. CAFs consist of three main subpopulations: myofibroblast-like (myCAFs), inflammatory (iCAFs), and antigen-presenting (apCAFs). MyCAFs are the most common subtype, with high α-SMA expression and a favorable tumor-restrictive role. iCAFs secrete numerous inflammatory and pro-tumorigenic cytokines, including interleukin (IL)-6. apCAFs express MHC class II and are therefore able to present antigens to CD4+ cytotoxic T lymphocytes [20].

## 4. Disregulation of VD in PDAC

It has been shown that pancreatic ductal cells in both normal and PDAC express the enzyme that catalyzes the conversion of 25(OH) D to 1, 25-dihydroxy VD. VDR has been detected in several pancreatic cell lines. Decreased VDR expression in pancreatic tumor tissues compared with normal pancreatic cells has been noted [20].

The VD system is deregulated in pancreatic diseases including chronic pancreatitis (CP) and PDAC. Hummel et al. [21] investigated the expression of VDR, 1,25-dihydroxyvitamin D3 24-hydroxylase (CYP24A1), and calcium-sensing receptor (CaSR), a VD target gene, in various pancreatic regions in patients with CP (*n* = 6) and PDAC (*n* = 17). This study showed that in CP, CYP24A1 expression was highest in endocrine pancreatic cells, while it was highest in PDACs in the transformed ducts. In patients with PDAC, CYP24A1 expression in the islets was significantly lower than that in patients with CP. These findings suggest that VD is deregulated in PDAC at two different points. In islets, CYP24A1 expression is decreased, which weakens the negative feedback regulation of VD-dependent insulin synthesis/secretion. In the transformed ducts, CYP24A1 expression is increased, which impairs the antiproliferative effect of VD in these cells [21].

## 5. Anti-Tumorigenic VD Impact on PDAC

**Figure 1:**
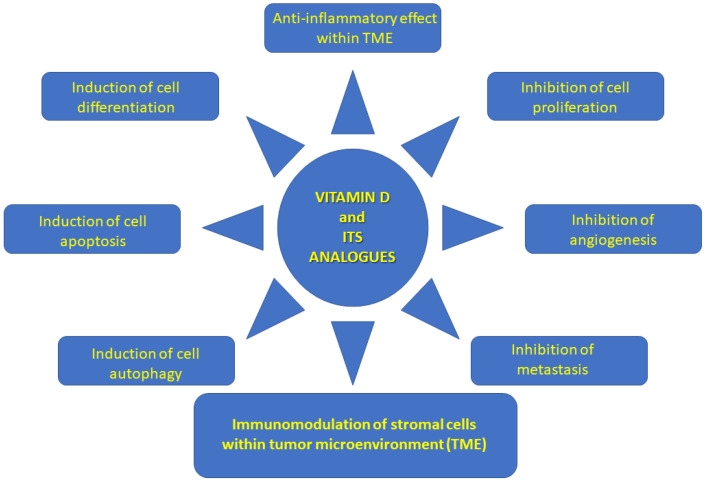
Anti-cancer role of VD and its analogs.

**Figure 2:**
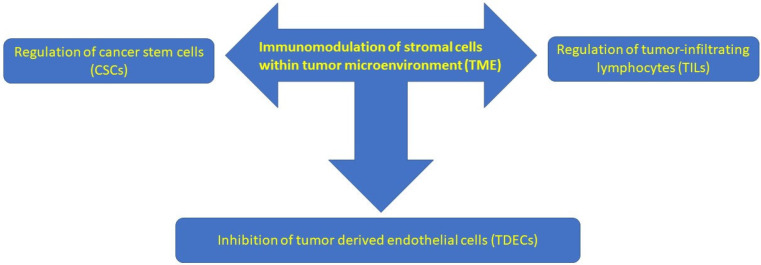
Impact of VD and its analogs on tumor microenvironment (TME).

Various mechanisms of VD and its analogs’ anti-tumorigenic effects on PDAC have been described in the worldwide literature, including the following: induction of kinase inhibitors depending on cyclin (p21, p27), stimulation of caspase-dependent apoptosis, synergism with chemotherapeutic drugs, inactivation of Hedgehog signaling, and alteration of insulin regulation. According to the literature, lower plasma 25(OH)D concentration is related to an increased PDAC risk in patients with diabetes [22,23]. VD and its analogs modulate PDAC stroma by activating VDR, attenuating pro-tumor signaling, increasing tumor vasculature, and decreasing fibrosis [24]. This is possible because VDR is expressed in PDAC stroma. Therefore, treatment with the VDR ligand calcipotriol decreases the markers of inflammation and fibrosis in pancreatitis and tumor stroma [25,26]. Transcriptional reprogramming of the tumor stroma has been shown to enable chemotherapeutic response and suggests VD priming as an adjunct in PDAC treatment. High VDR expression has been reported in PSCs. Stromal VDR activation overcomes chemotherapeutic drug resistance. The VDR ligand plus gemcitabine enhanced survival in a PDA mouse model [25]. CAFs activate tumor invasion and metastasis, proliferate, and resist PDAC cell death, while cancer stem cells (CSCs) and tumor-initiating cells (TICs) play a role in tumorigenesis, progression, metastasis, and tumor recurrence. VD regulates tumor angiogenesis, progression, and metastasis by modulating the gene signature of the above-mentioned tumor stromal cells [27]. The immunomodulatory impact of VD on PDAC TME is presented more fully in Section 2. The fibrotic tumor stroma, which creates an environment that promotes tumor growth, is resistant to chemotherapy and other anti-cancer drugs. It has been suggested that TME is one of the main barriers to successful systemic PDAC [27]. Therefore, remodeling of the tumor stroma by VD and its analogs (via VDR) can decrease systemic anti-cancer drug resistance. Degradation of the TME seems to be the key to successful systemic therapy for PDAC. Therefore, there are numerous studies on the pleiotropic effects of VD analogs in systemic anti-cancer therapy in PDAC [27]. The anti-cancer role of VD and its analogs, including their impact on the TME, is presented in Figure 1 and Figure 2 [27].

## 6. Optimal Methods for Assessment of VD Status in the Human Body and Increasing Serum 25(OH) D Concentration

**Figure 3:**
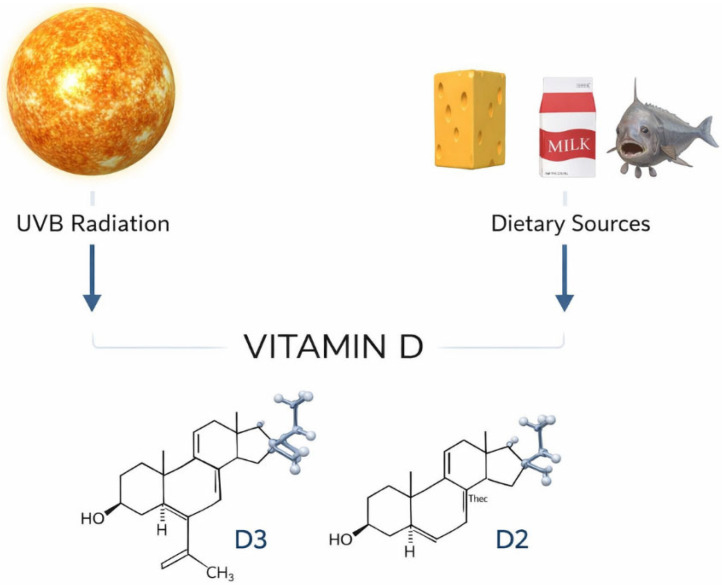
Two main sources of VD in the human body: sunlight exposure and diet including fatty saltwater fish, eggs, milk and milk products.

There are two main sources of VD in humans. Cholecalciferol (VD3) is generated in the skin after exposure to ultraviolet B (UVB) light via photolysis of cutaneous 7-dehydrocholesterol. Food (such as fatty saltwater fish, eggs, milk, and milk products) is the second most common source of VD in humans. Animal source products contain VD 3, while products of some plant and fungi sources contain ergocalciferol (VD 2). Food contains an inactive form of VD that is transported in the bloodstream by VD-binding proteins (VDBP) to the liver, where it is hydroxylated by sterol 27-hydroxylase (CYP27A1) and converted to 25-hydroxycholecalciferol (25(OH)D), which is the most stable VD metabolite. It has a serum circulation half-life of 15 days [6]. The main sources of VD are shown in Figure 3.

Regarding the production of VD following sunlight exposure, according to some authors, living in areas characterized by higher sun exposure is related to decreased mortality rates for PDAC in people from the Caucasus [28,29,30], Japan [31,32], and Africa and America [33]. In addition, age, obesity, and African American ethnicity, related to decreased serum 25(OH)D concentration, are associated with an increased PDAC risk. Therefore, a correlation between increased serum 25(OH)D concentration and decreased PDAC risk has been reported [34].

The serum concentration of 25-hydroxycholecalciferol, the most stable VD metabolite, is a useful biomarker to assess VD status in the human body [35,36]. A consensus concerning an adequate serum 25(OH)D concentration has not yet been developed. The different definitions of serum 25(OH)D concentrations are presented in Table 1. Ranges of serum 25-hydroxyvitamin D concentrations reported in Table 1 reflect commonly used reference intervals; however, these ranges may not represent optimal levels with respect to pancreatic cancer risk and should be interpreted with caution, particularly across populations with differing genetic backgrounds, baseline vitamin D exposure, and metabolic adaptations, as discussed in the Section 9 [35].

Intense sun exposure can cause skin damage and neoplasms. Therefore, caution is warranted when acquiring VD using sunlight. As discussed above, food is the second source of VD, but only a few foods contain substantial amounts of VD. Therefore, direct supplementation with VD3 at doses of 800–4000 international units (IU)/day (20–100 µg/day) is commonly recommended for serum 25(OH)D concentrations of 30–60 ng/mL (75–150 nmol/L) [35].

## 7. Methods of the Literature Search

This is a narrative review. The PubMed and Web of Science databases, including papers published in the period from 1985 to 2025, have been reviewed. The search terms and mesh heading were as follows: “pancreatic cancer” or “pancreatic ductal adenocarcinoma” and “vitamin D” or “vitamin D3” as well “vitamin D” and “immune status.” Selected articles on the relationship between VD and PDAC risk are described in this review. English-language, full-text original articles, including pre-clinical (experimental) and clinical (meta-analyses, prospective randomized, epidemiological) studies, were included in the further analysis. The literature review was organized according to the type of inquiry into VD (UVB exposure, VD intake, evaluation of VD status, and genetic analysis). The experimental (pre-clinical) studies were followed by the clinical studies. The meta-analyses were followed by prospective randomized and retrospective studies. Epidemiological studies were presented in a separate section.

## 8. Literature Review

### 8.1. Pre-Clinical Experimental Studies on the Association Between VD and PDAC

Several studies have shown that VD and its analogs inhibit the proliferation rate of pancreatic cancer cell lines. It has been shown that 1,25 VD analogs inhibit pancreatic cancer cell proliferation, induce differentiation, and promote apoptosis in vitro [5,37,38,39,40,41,42,43,44]. Zugmaier et al. [38], Pettersson et al. [39], Albrechtsson et al. [40], Ohlsson et al. [41], and Cheng et al. [44] assessed the impact of VD analogs on PDAC lines. The authors demonstrated inhibition of the growth of PDAC cells by the above-mentioned substances, such as VD and VD analogs, including retinoids. Retinoids (active forms of fat-soluble vitamin A) and 1,25-dihydroxyvitamin D3 play important roles in maintaining cellular functions in humans. There are various forms of retinoid, such as retinol and its natural metabolites or analogs, including all-trans retinoic acid (ATRA), 9-cis retinoic acid (9-cis RA), and 13-cis retinoic acid (13-cis RA). These retinoids are involved in several important functions including gene regulation, cellular development, differentiation, proliferation, and apoptosis in human epithelial cells [44]. In a recent study by Cheng et al. [44], human PDAC cells were treated with Tumor Necrosis Factor—alpha (TNF-α) in the presence or absence of 13-cis RA and VD3. The authors demonstrated significant inhibition of PDAC cell invasion mediated by TNF-α by VD3 and 13-cis retinoic acid [44].

Ohlsson et al. [41] analyzed the impact of other fat-soluble vitamins on pancreatic cancer cell lines. This study showed that vitamins A and D, but not E and K, decreased the number of human pancreatic cancer cell lines. High concentrations of vitamin A and D analogs decreased the number of PDAC cells. There was no significant effect of the combination of the two vitamins [41].

Gorchs et al. [43] and Moz et al. [5] analyzed the role and mechanism of action of a VD3 analog (calcipotriol) on PDAC cells. Gorchs et al. [43] investigated the influence of the VD3 analog (calcipotriol) on the activation of human pancreatic CAFs and T cells using two- and three-dimensional (2D, 3D) cell culture models. The authors observed a decrease in CAF proliferation and migration, as well as decreased secretion of the pro-tumorigenic prostaglandin E2, interleukine-6, periostin, and leukemia inhibitory factor caused by the investigated VD analog. In addition, a decrease in T-cell proliferation and production of IFN-γ, granzyme B and interleukine-17, as well as an increase in interleukine-10 secretion, caused by the above-mentioned VD analog was noted [43]. Moz et al. [5] showed that calcipotriol decreased spontaneous and induced PDAC TNF-α secretion by peripheral blood mononuclear cells (PBMCs), as well as decreased intracellular transforming growth factor beta (TGF-beta). In addition, it antagonizes the alterations in immune cells induced by PDAC cells [5].

The complex mechanism of the impact of VD or VD analogs on PDCA lines has been analyzed in some studies: [3,45] assessed the regulation of proteins involved in apoptosis in pancreatic cancer cells. The VD analog, EB1089, was used in this study. The study showed a decrease in apoptosis induced by 9-cis retinoic acid by the investigated VD analog in PDAC cells. This observation was associated with decreased p27Kip1 protein levels [42]. Li et al. [3] analyzed the effects of VD3 levels on the expression of VDR and cell cycle-related proteins CDKN1A (p21) and CDK1 in pancreatic cells and Panc-1 PDAC cells. This analysis showed that increased VD3 concentrations upregulated VDR, CDKN1A, and CDK1 in normal pancreatic cells. However, this has not been reported for advanced metastatic PDAC cells [3]

The most important studies mentioned above are summarized in Table 2.

### 8.2. Clinical Studies on the Association Between VD and PDAC

#### 8.2.1. The Role of Sunlight or Ultraviolet B (UVB) Irradiance and VD in PDAC

Mohr et al. [46] demonstrated a higher PDAC risk in participants living in higher-latitude areas (*p* < 0.001). There was a significant and independent inverse relationship between UVB irradiance and PDAC incidence in males (*p* < 0.01) and females (*p* = 0.02). Incidence rates were 50% lower in countries with serum 25(OH)D levels >30 ng/mL (75 nmol/L) than in those with VD concentrations ≤30 ng/mL. Therefore, the authors concluded that higher incidence rates of PDAC were reported in areas characterized by lower UVB irradiance [46].

Another study by Kinoshita et al. [31] showed that low solar radiation and low temperature may be associated with a higher PDAC risk [31]. Kato et al. [47] reported higher mortality due to PDAC in northern Japan, Scandinavia, and other northern European countries. In addition, the authors observed a strong positive correlation between mortality and latitude within Japan, with correlation coefficients of 0.612 (men) and 0.615 (women). International comparisons also showed a positive correlation between mortality and latitude, with correlation coefficients of 0.724 (men) and 0.725 (women). Moreover, there was a negative correlation between mean temperature and mortality caused by PDAC, both in Japan (correlation coefficients of −0.587 in men and −0.630 in women) and internationally (correlation coefficients of −0.773 in men and −0.729 in women). The change in latitude is thought to influence climatic factors such as sunlight and temperature [47]. The above-mentioned studies showed a relationship between UVB irradiation and PDAC risk and prognosis. Generally, higher latitudes are associated with lower temperatures related to higher PDAC risk and cancer-specific mortality [47]. Boscoe et al. [29] also reported an inverse relationship between UVB irradiance and PDAC incidence and mortality in an American population [29].

Similarly, in a study by Garland et al. [48], the PDAC incidence rate was six times higher in participants living in areas characterized by low UVB irradiance than in patients in areas characterized by high UVB irradiance [49]. This study demonstrated an inverse association between cloud-adjusted UVB irradiance and PDAC incidence. This result is consistent with an inverse association between overall VD deficiency in geographical areas characterized by lower UVB irradiance and PDAC risk [48]. In a retrospective study by Eryilmaz et al. [48], a comparative analysis of the impact of sunlight exposure on the prognosis of PDAC patients in two areas characterized by different sunlight exposures was performed. This study showed better OS in patients with PDAC residing in regions with greater sunlight exposure [48].

In most epidemiological studies, ecologic adjustments for various confounding factors (such as smoking, outdoor occupation, and food) were made, but according to the authors, they were not optimal, relying on proxy measures, survey data, spatial interpolations, and other imperfect instruments. These factors can affect the final results [29].

Although excessive exposure to UVB may have an adverse impact on human health (it is a risk factor for malignant skin neoplasms), moderate sunlight exposure triggers VD production. Considering the immune and anti-cancer effects of VD, solar radiation could be beneficial in the prevention of PDAC and in improving the prognosis of PDAC patients (decreases mortality).

The most important studies mentioned above are summarized in Table 3.

#### 8.2.2. The Role of VD Oral Intake in PDAC Prevention and Treatment

A meta-analysis by Liu et al. [49] showed that VD consumption moderately decreased the PDAC incidence. According to the authors, daily consumption of 10 μg of VD may lead to a 25% reduction in PDAC incidence [49].

In another meta-analysis by Genkinger et al. [50] involving 14 prospective cohort studies, including 2212 PDAC patients, the authors investigated the association between specific foods (as sources of VD) and PDAC risk. This study did not show a relationship between total milk dietary consumption and PDAC risk. In addition, there was no association between PDAC risk and dietary consumption of whole milk or low-fat milk, as well as other milk products. Moreover, there was no relationship between dietary and total VD consumption and PDAC risk [50].

In another prospective study by Skinner et al. [8], higher VD intake compared to lower VD consumption (<150 IU/d) was associated with lower PDAC risk. According to the authors, stronger associations are possible in males than in females [8].

Waterhouse et al. [51] demonstrated a significant association between VD consumption and higher PDAC risk with VD dietary consumption. An increase in dietary VD consumption of 100 IU/day was related to a 13% increase in PDAC risk [OR = 1.13, 95%CI: 1.07–1.19; *p* < 0.05) [52].

The most important studies mentioned above are summarized in Table 4.

#### 8.2.3. The Association Between VD Serum Concentration (Evaluation of VD Status) and PDAC

##### Inverse Association

Wolpin et al. [53] noted a lower serum 25(OH)D concentration in PDAC patients than in controls (61.3 versus 64.5 nmol/L, *p* = 0.005). Logistic regression analysis revealed an inverse association between the serum 25(OH)D concentration and PDAC risk. Therefore, this study showed that increased serum 25(OH)D concentrations were associated with lower PDAC risk [53]. Yuan et al. [13] reported longer OS in PDAC patients with sufficient baseline serum 25(OH)D concentrations than in patients with VD deficiency [13]. In a retrospective study by Cho et al. [52], a serum 25(OH)D concentration of <20 ng/mL was associated with poor prognosis in PDAC patients with stage III and IV [52].

##### No Association

A meta-analysis by Liu et al. [53] showed that there was no significant association between 25(OH)D concentration and PDAC risk. In this study, increased VD concentrations were associated with a 1.14-fold increase in PDAC risk [53]. Another meta-analysis by Zhang et al. [54] showed no significant inverse relationship between high serum 25(OH)D level and PDAC mortality. However, there was no significant inverse association between high serum 25(OH)D concentration, VD consumption, and PDAC risk [54]. The study by Stolzenberg-Solomon et al. [55] showed that very high serum 25(OH)D levels (≥100 nmol/L) were associated with an increased risk of pancreatic cancer, particularly at latitudes 35° N–42° N (moderate to high UVB exposure), with an odds ratio of 2.12 (95% CI: 1.23–3.64). These findings suggest that recommendations to raise VD levels in healthy individuals for cancer prevention should be approached with caution [55]. Similarly, Helzlsouer et al. [56] observed that both deficient and very high VD concentrations (>124 nmol/L) were associated with increased mortality. The Cohort Consortium Vitamin D Pooling Project of Rarer Cancers (VDPP), including 10 prospective cohorts from the US, Finland, and China, examined associations between serum 25(OH)D and seven less common cancers, including pancreatic cancer. No clear protective effect was observed, and high 25(OH)D levels were linked to increased pancreatic cancer risk. In women, both low and very high concentrations (>124 nmol/L) were associated with higher mortality, suggesting that any protective effect of vitamin D is confined to a relatively narrow optimal range, and that caution is needed with high-dose supplementation [56]. These data indicate that the relationship between VD and pancreatic cancer risk is likely U-shaped, with a narrow optimal range for protective effects [55]. In van Duijnhoven et al.’s study [57], there was no inverse association between increased serum 25(OH)D levels and PDAC risk [57]. McGovern et al. [58] assessed the relationship between 25(OH)D concentration and time to progression (TTP) and OS. In this study, there was no association between serum 25(OH)D concentration and TTP or OS [58].

##### Positive Association

Some authors have demonstrated that a higher PDAC is related to a higher VD status. In a prospective case–control study called the Alpha-Tocopherol, Beta-Carotene Cancer Prevention Study (ATBC), performed on a cohort of male Finnish smokers by Stolzenberg-Solomon et al. [9], higher serum 25(OH)D concentrations were significantly associated with a threefold increase in PDAC risk (*p* = 0.001). In addition, serum retinol concentrations were analyzed. There is an inverse association between serum retinol levels and PDAC risk [9]. Similarly, a case–control study by Stolzenberg-Solomon et al. [59] performed a pooled nested case–control study using participants from eight cohorts within the Cohort Consortium Vitamin D Pooling Project of Rarer Cancers (VDPP, 1974–2006) to assess whether pre-diagnostic circulating 25(OH)D levels were associated with pancreatic cancer development. During a median follow-up of 6.5 years, 952 incident pancreatic adenocarcinoma cases were identified. Controls (*n* = 1333) were matched to cases by cohort, age, sex, race/ethnicity, date of blood collection, and follow-up duration. Conditional logistic regression was applied to estimate odds ratios (ORs) and 95% confidence intervals (CIs), adjusted for smoking, body mass index, and diabetes. Serum 25(OH)D levels were categorized using clinically relevant cutpoints, with 50–<75 nmol/L as the reference group. No significant associations were observed among participants with lower 25(OH)D levels; however, high 25(OH)D concentrations (≥100 nmol/L) were associated with a statistically significant twofold increase in pancreatic cancer risk (OR = 2.12; 95% CI: 1.23–3.64). These findings also indicate that recommendations to raise vitamin D levels in healthy individuals for cancer prevention should be considered with caution [59].

#### 8.2.4. Association Between VD Concentration and Survival in Patients with PDAC

A recent meta-analysis by Shen et al. [60], involving 16 studies including 529,917 participants, revealed a positive association between VD serum concentration and survival in PDAC patients. This study suggested the association between high circulating VD concentrations and decreased mortality in PDAC. It should be pointed out that the same study reported the lack of association between high VD serum concentrations and PDAC incidence [60].

Loon et al. [10] investigated the associations between serum 25(OH)D concentration and progression-free survival (PFS) and OS in patients with PDAC. This finding revealed that VD deficiency was more frequently noted in patients with newly diagnosed advanced PDAC. Significantly lower 25(OH)D concentrations were reported in black patients than in white patients. There was no association between baseline VD concentrations and PFS/OS in patients with advanced PDAC receiving gemcitabine. Thus, this study suggests that VD has a limited influence on survival in advanced PDAC [10].

A prospective biomarker BIOPAC study by Rassmussen et al. [61], based on a prospective database, showed an association between pretreatment serum 25(OH)D concentrations, inflammatory biomarkers (CRP, IL-6, and YKL-40), and OS in PDAC patients. In this study, VD deficiency was associated with increased concentrations of inflammatory biomarkers at all PDAC stages. A positive association between VD concentrations and OS was noted in patients with PDAC in resected stages I and II, but not in patients with advanced PDAC [61].

A meta-analysis by Li et al. [62] showed an association between serum 25(OH)D concentration and OS (hazard ratio = 2.37; 95% confidence interval, 2.22–2.54; *p* <0.001), but not PFS, in patients with advanced PDAC. This meta-analysis involved seven studies on 2369 patients. The authors pointed out significant heterogeneity between studies regarding OS. This high heterogeneity could be attributed to the studies’ different regions, designs, sample sources, and detection methods of 25(OH)D. In addition, Begg’s and Egger’s tests indicated the presence of publication bias. Despite these limits, this is the first meta-analysis to assess the association between 25(OH)D concentrations and OS in patients with PDAC [62].

The most important studies mentioned above are summarized in Table 5.

### 8.3. Genetic Analysis

A case–control Ontario Pancreas Cancer Study by Anderson et al. [64] showed potential associations between polymorphisms in VD-related pathway genes and PDAC risk. A significant association between single-nucleotide polymorphisms (SNPs) in CYP24A1, CYP2R1, calcium-sensing receptor (CASR), VD-binding protein (GC), retinoid X receptor-alpha (RXRA), and LRP2 genes and PDAC risk has been reported. There was an inverse association between CYP2R1 rs10741657 and PDAC risk and a positive association between CYP24A1 rs6127119 and PDAC risk. There was no significant relationship between the CUBN, CYP27B1, DHCR7, and NADSYN1 and PDAC risk [64].

Piper et al. [65] analyzed the association between VD-binding protein (DBP) and PDAC risk in an American population. This study found no significant association between DBP concentration and PDAC risk. Comparisons of various serum 25(OH)D concentrations revealed a significantly higher PDAC risk in patients in the patients with the highest VD concentration (≥100 nmol/L; OR = 3.23; 95%CI: 1.24–8.44). However, in patients in the lowest group (<25 nmol/L; OR = 2.50; 95% CI: 0.92–6.81), a relationship between 25(OH)D levels and PDAC risk was not observed. Therefore, this study did not confirm the protective role of DBP or serum 25(OH)D concentration on PDAC risk [65].

The most important studies mentioned above are summarized in Table 6.

### 8.4. Optimal VD Concentrations for Reduction in PDAC Risk and Improving Survival in PDAC Patients

Although there are numerous studies regarding the association between VD and PDAC, there is no consensus regarding the adequate serum 25(OH)D concentration to prevent cancer and improve survival in cancer patients. Based on the results of the meta-analyses, plasma VD concentrations of 54–135 nmol/L are recommended to decrease cancer mortality. Another study recommended a VD concentration of 100–125 nmol/L (40–50 ng/mL). There are recommendations for oral VD supplementation of 4000 IU/day or 2000 IU/day plus exposure of 50% of the body surface to sunlight for 12 min every day in order to achieve a 25(OH)D level of 125 nmol/L [6,66].

Both supplementation and high doses of VD have been limited because of the risk of hypercalcemia. Recently, numerous synthetic VD analogs that do not affect calcium concentration but have antiproliferative and immunomodulatory roles have been introduced [6,67]. Few clinical studies have investigated the role of these VD analogs in PDAC prevention and treatment. Therefore, further studies are required to optimize the VD doses and introduce them in clinical practice [6].

### 8.5. Vitamin D as Adjunct Management with Other Anti-Cancer Treatment

As mentioned above, the PDAC TME is the main cause of resistance to current anti-cancer therapy. The immunomodulatory effect of VD on various components of PDAC TME can be used as a novel treatment for PDAC. In this management strategy, the pleiotropic VD effect on standard established chemotherapy is used. There are a limited number of clinical studies assessing the efficacy of the combination of VD or its analogs with established chemotherapy in the treatment of PDAC [11,25,68,69,70].

In a study by Yu et al. [68], in a human pancreatic cancer model, the authors reported that the VD analog, calcitriol, used with gemcitabine, promoted caspase-dependent apoptosis that caused increased anti-tumor activity compared to either agent alone. Thus, calcitriol promotes the antiproliferative effect of gemcitabine [68].

In another study by Sherman et al. [25], in a murine cell model, the combination of gemcitabine and calcipotriol significantly reduced tumor volume, with transient or sustained reduced tumor growth noted in approximately 70% of mice. In addition, a reduction in tumor-associated fibrosis was noted in mice receiving combination therapy compared with that in controls [25].

A study by Mukai et al. [69], conducted on 86 patients with advanced PDAC who underwent resection with or without preoperative chemoradiotherapy (CRT), showed that a larger number of α-SMA-positive CAFs was a significant risk factor for distant metastasis-free survival (DMFS), regardless of CRT, the increased number of CAFs in patients after preoperative CRT, or with a lower serum VD concentration. In this study, higher plasma VD concentrations were associated with longer DMFS in patients with PDAC after preoperative CRT. Moreover, stromal PDAC cells activated by radiotherapy were inhibited by VD supplementation, and VD supplementation inhibited activation [69].

Kang et al. [70] designed 57 nonsecosteroidal VDR modulators based on the phenyl-pyrrolyl pentane skeleton. The authors reported inhibition of PSCs and suppression of the interaction between cancer cells and PSCs in vitro by compounds C4, I5, and I8, as well as anti-tumor activity without hypercalcemia, using compound I5 combined with gemcitabine [70].

The most important studies on supplementation of VD or VD analogs with concurrent established chemotherapy in PDAC are summarized in Table 7. Few studies have investigated the combination of VD or VD analogs with anti-cancer immunotherapy and viroimmunotherapy [6,24,26]. Currently, several clinical trials on the combination of VD analogs and chemotherapy are ongoing [6].

## 9. Discussion

### 9.1. VD and PDAC: Overview of Evidence

PDAC remains a malignancy with poor prognosis and limited therapeutic options, prompting investigation into modifiable risk factors and adjunctive therapies. VD, a secosteroid hormone with pleiotropic effects on cellular proliferation, differentiation, and immune modulation, has been extensively studied in the context of cancer prevention and treatment [6,71].

There are not a lot of studies regarding the association between VD and PDAC in the literature data. The most important studies mentioned above are summarized in Table 2, Table 3, Table 4, Table 5, Table 6 and Table 7. Synthesis of the above-mentioned studies is presented in Table S1. The limitations of the above-mentioned studies are summarized in Table S2. The future perspectives are presented in Table S3.

The findings of pre-clinical experimental studies show that VD might counteract PDAC, but the results of clinical trials regarding the association between VD and PDAC are inconsistent. They included trials on the association between UVB irradiance, oral VD intake, serum VD concentration, and genetic analysis.

This analysis indicates that studies evaluating VD status based on serum concentration of 25(OH)VD are the most objective, because in epidemiological trials assessing the association between sunlight or VD intake, various confounding factors (such as lifestyle, diet, daily outdoor activity, geographical region, liver and kidney condition, other supplements, and drugs) can affect the final results.

This literature review has shown that studies concerning the association between VD status and PDAC are the most numerous but also the most contradictory in the literature. According to most authors, VD is associated with a lower PDAC risk and improved survival in patients with PDAC, but according to other authors, higher VD concentrations are related to higher PDAC risk. According to some authors, there is no significant association between VD and PDAC risk or survival in PDAC patients. Regarding ways of obtaining VD, both sunlight and oral intake sources have limitations and may lead to adverse health effects. Excessive UVB irradiation is associated with a higher risk of developing malignant skin neoplasms. However, excessive oral VD supplementation can lead to hypercalcemia. Therefore, further prospective randomized and large epidemiological studies regarding the impact of controlled oral VD supplementation as well as sunlight exposure, taking into account other risk factors (such as smoking and obesity) on PDAC risk and survival, are needed. Therefore, caution should be exercised before recommendations concerning increasing 25(OH)D concentrations for cancer prevention in both healthy patients and patients with PDAC [16].

### 9.2. Limitations of the Presented Studies

There are some limitations to the interpretation of the results of the aforementioned studies. First, most of the publications regarding the association between VD and PDAC risk are case–control studies. No prospective randomized controlled trials have been conducted in the literature. In addition, only four large meta-analyses on this topic have been published to date. None of the studies showed a significant inverse association between VD and PDAC risk or survival in patients with PDAC. Moreover, the presented studies were performed in different geographical areas with different sunlight exposure levels, which could impact the final results. Outdoor activity (impacting sunlight exposure) and diet (types and amounts of food and oral VD supplementation), as well as oral VD supplementation (type of drug and VD amount in the drug), as the two main sources of VD in the human body, should be adjusted in the multiple regression analysis to determine the relationship between VD and PDAC risk. Regarding the impact of VD on survival in patients with PDAC, numerous confounding factors that could impact the final results should be included in the multivariate Cox regression analysis, such as surgical margins (R0/R1/R2), vascular invasion, peripheral nerve invasion, tumor clinical staging, tumor histopathological differentiation, and serum CA19-9 concentrations [68]. Because of the essential role of the liver and kidneys in VD metabolism, other biochemical parameters, including serum alanine transaminase (ALT), aspartate aminotransferase (AST), gamma-glutamyl transferase (GGT), alkaline phosphatase (ALP), bilirubin, and creatinine serum concentrations, as indicators of liver and kidney failure, should be considered in the Cox regression analysis [72,73].

Pre-clinical experimental studies have demonstrated a positive anti-cancer effect of VD on PDAC cell lines, including inhibition of PDAC cell proliferation, induction of cell differentiation, and promotion of cancer cell apoptosis. Data from epidemiological studies have reported contradictory results, suggesting both positive and negative associations, as well as no association between VD and PDAC risk. Therefore, based on the results presented in this review article, VD or its analogs can be considered as economical agents in combination with chemo- or immunotherapy for PDAC treatment; however, we cannot strongly recommend higher doses than the current standard of oral VD and more extensive sunlight exposure to reduce PDAC risk. Further investigation is required to answer these questions regarding the relationship between VD and PDAC. Therefore, caution is warranted before the introduction of recommendations to use higher than standard doses of VD in PDAC prevention and treatment. This analysis indicates that VD supplementation in PDAC prevention and treatment should be considered only after large prospective randomized controlled trials and meta-analyses confirming its beneficial impact on PDAC risk and survival. It is important to emphasize investigations of VD analogs that have anti-tumorigenic effects, but do not lead to hypercalcemia.

### 9.3. The Association Between Increased VD Intake and Higher Cancer Risk

The other important issue, which should be pointed out in this review, is the association between increased VD intake and higher cancer risk. The reported associations between increased VD intake and higher cancer risk are not related to VD toxicity but rather reflect specific study populations and cancer types. In a large Danish cohort study, higher vitamin D levels were associated with increased incidence of non-melanoma skin cancer (HR 1.09 per 10 nmol/L increment), melanoma (HR 1.10), prostate cancer (HR 1.05), and hematological cancers (HR 1.03), though these associations were observed across the normal physiological range rather than at toxic levels [74]. These findings likely reflect confounding by sun exposure for skin cancers and detection bias in populations undergoing more frequent medical surveillance, rather than a causal harmful effect of VD itself [74].

Importantly, the preponderance of evidence demonstrates protective or neutral effects of VD across most cancer types. Meta-analyses of randomized controlled trials show that VD supplementation at doses up to 4800 IU daily (achieving serum levels of 54–135 nmol/L or 22–54 ng/mL) does not increase cancer incidence (RR 0.98, 95% CI 0.93–1.03) and significantly reduces cancer mortality (RR 0.87, 95% CI 0.79–0.96) [2]. Even at serum 25(OH)D levels exceeding 100 nmol/L (40 ng/mL), no increased cancer risk was observed [75]. The 2024 Endocrine Society Clinical Practice Guideline found that high-dose VD supplementation (defined as doses substantially above standard recommendations) was associated with increased all-cause mortality (RR 1.22, 95% CI 1.06–1.39), but this was not specifically attributed to cancer and may relate to other adverse effects [76]. According to this viewpoint, true VD toxicity (typically occurring at serum levels > 150 ng/mL or 375 nmol/L) manifests primarily as hypercalcemia and related complications, not increased cancer risk [77,78].

The associations vary by population subgroups: observational studies suggest protective effects are strongest in normal-weight individuals, with attenuated benefits in those with obesity [79,80]. Additionally, the dosing strategy matters—daily supplementation appears more beneficial than intermittent bolus dosing for cancer mortality reduction [75,79,81].

### 9.4. Ethnic Differences in VD Metabolism and PDAC Risk

Ethnic and geographic variability in VD metabolism represents an important source of heterogeneity in studies evaluating the association between circulating 25-hydroxyvitamin D [25(OH)D] concentrations and PDAC risk. Populations with darker skin pigmentation or those residing at high latitudes generally exhibit lower serum 25(OH)D levels, largely due to reduced ultraviolet B-mediated cutaneous synthesis. Serum 25(OH)D levels are naturally lower in darker-skinned populations, apparently because less UVB enters the skin to synthesize this vitamin. Serum levels are also naturally lower in populations that live at high latitudes and receive much less UVB, notably the Inuit of Alaska, northern Canada, and Greenland, and the Arctic peoples of northern Eurasia [82,83,84]. However, accumulating evidence indicates that these lower circulating concentrations may reflect physiological adaptation rather than true VD deficiency. Studies conducted among Inuit and other Arctic populations have demonstrated more efficient intestinal calcium absorption [85,86,87], increased conversion of 25(OH)D to its biologically active metabolite 1,25-dihydroxyvitamin D [86], and population-specific differences in VD-binding protein structure and affinity [88,89]. Similar adaptive mechanisms have been reported in individuals of African ancestry. If the lower serum 25(OH)D concentrations observed in these populations reflected true vitamin D deficiency, no homeostatic mechanism would be expected to maintain them at reduced levels. However, evidence from studies of African Americans with varying degrees of African ancestry suggests genetically determined regulation of vitamin D homeostasis, as both sunlight exposure and dietary intake were approximately 46% less effective in increasing serum 25(OH)D concentrations in individuals with more African ancestry compared with those with less African ancestry [90]. Importantly, despite lower serum 25(OH)D levels, African American populations display higher bone mineral density and lower fracture risk compared with European Americans [91,92,93], and adolescent females demonstrate higher calcium retention and bone formation efficiency [94]. Further, participants in the Shanghai and MEC studies, who lived at low latitudes, tended to have lower 25(OH)D concentrations overall (i.e., Shanghai), or because of race/ethnicity (e.g., African Americans). As a result, few participants residing at low latitudes had 25(OH)D concentrations greater than 100 nmol/L [59]. These observations support the notion that serum 25(OH)D thresholds derived predominantly from European populations may not be universally applicable. Accordingly, ethnic background and population-specific VD metabolism should be considered when interpreting associations between VD status and PDAC risk, as also reflected in large cohort analyses reporting substantial variation in 25(OH)D distributions across ethnic groups [59]. Among men, well-established risk factors—primarily cigarette smoking and diabetes mellitus—account for nearly all of the disparity in incidence between Black and White populations. Among women, however, additional factors seem to play a role in this racial difference, particularly moderate to heavy alcohol use and higher body mass index. Without these influences, pancreatic cancer incidence among Black individuals would likely not be higher than that observed among White men or women [95]. Failure to account for such population-specific adaptations in VD metabolism may partly explain inconsistent associations between circulating 25(OH)D concentrations and PDCA risk observed across epidemiological studies.

### 9.5. Potential Adverse Effects of High VD Levels

Another important consideration in evaluating the role of VD in pancreatic cancer concerns the potential adverse effects associated with high circulating VD concentrations. Although classical VD toxicity—characterized by hypercalcemia and related complications—typically occurs only at markedly elevated serum 25(OH)D levels, several epidemiological studies have reported increased cancer risk within ranges not considered toxic. In the Alpha-Tocopherol, Beta-Carotene Cancer Prevention Study, higher serum 25(OH)D concentrations were associated with a significantly increased PDAC risk among male smokers [59]. Similar concerns have been raised in pooled analyses and meta-analyses suggesting that very high VD status does not confer additional protective effects and may, in some contexts, be associated with adverse outcomes [53,56,96]. These findings have led to the hypothesis of a non-linear or U-shaped relationship between VD status and cancer risk, whereby both low and high circulating concentrations may be unfavorable. Potential mechanisms proposed include dysregulation of calcium signaling, altered immune modulation, and interactions with genetic variability in VD transport and receptor pathways, including differences in VD-binding protein concentrations [65]. Collectively, these findings suggest that any potential protective effect of vitamin D with respect to pancreatic cancer, if present, is likely confined to a relatively narrow range of circulating 25(OH)D concentrations, and that levels ≥100 nmol/L should not be assumed to be universally safe, despite falling within ranges traditionally considered normal. Importantly, these associations appear to be strongly influenced by population characteristics, smoking status, baseline VD exposure, and genetic background. Consequently, current evidence does not support the routine pursuit of high serum 25(OH)D concentrations for cancer prevention and underscores the need for cautious interpretation of observational findings when formulating clinical or public health recommendations.

In conclusion, currently, there is not sufficient evidence to recommend supplementation of VD or its analogs for PDAC patients. This analysis indicates that its use should remain limited to research settings.

## 10. Summary and Conclusions

Despite compelling pre-clinical evidence supporting anti-tumorigenic effects of VD in PDAC, its clinical relevance remains uncertain. Experimental studies consistently demonstrate inhibitory effects of VD on PDAC cell proliferation, induction of differentiation, modulation of the tumor microenvironment, and immune regulation. However, translation of these findings into clinical benefit has proven challenging.

Epidemiological and clinical studies evaluating the association between VD status and PDAC risk or outcomes yield inconsistent results. Observational studies report heterogeneous associations between circulating 25-hydroxyvitamin D [25(OH)D] concentrations and PDAC incidence or survival, while evidence derived from dietary intake and supplementation studies is similarly inconclusive. Some pooled analyses suggest improved survival at moderate VD levels, whereas others report null associations or increased risk at higher concentrations. Large randomized controlled trials have not demonstrated a clear preventive or therapeutic benefit of VD supplementation, likely reflecting methodological heterogeneity, inclusion of VD-replete populations, variability in dosing regimens, and substantial interindividual differences in VD metabolism.

Importantly, emerging evidence suggests that both low and high circulating 25(OH)D concentrations may be unfavorable, supporting a non-linear relationship between VD status and cancer risk. Population-specific differences in VD metabolism, genetic background, baseline exposure, obesity, smoking status, and comorbidities further complicate interpretation and may partly explain discrepant findings across studies.

Overall, the current evidence is insufficient to support VD supplementation beyond standard recommendations for PDAC prevention or treatment. The available data should be regarded as hypothesis-generating rather than practice-changing, and routine use of high-dose VD supplementation for PDAC cannot be recommended at present. Future research should prioritize well-designed, adequately powered randomized trials with careful stratification by baseline VD status, standardized assessment of 25(OH)D concentrations, and consideration of genetic and metabolic variability. Exploration of VD analogs with improved safety profiles and evaluation of VD as an adjunct to established systemic therapies may further clarify its potential role in PDAC management.

## Figures and Tables

**Table 1:** Definitions of the 25(OH)D concentrations according to the Food and Nutrition Board of the Institute of Medicine and the Endocrine Society [6,36,37].

Definition	25(OH)D Concentrations
**The Food and Nutrition Board of the Institute of Medicine** [30]	
Deficiency	<30 nmol/L (12 ng/mL)
Inadequacy	30–50 nmol/L (12–20 ng/mL)
Normal	50–125 nmol/L (20–50 ng/mL)
High	>125 nmol/L (50 ng/mL)
**The Endocrine Society** [37]	
Deficiency	<50 nmol/L (20 ng/mL)
Insufficiency	52–72 nmol/L (21–29 ng/mL)

**Table 2:** A summary of the most important pre-clinical (experimental) studies on the association between vitamin D and pancreatic cancer.

Pre-Clinical (Experimental Studies) on the Role of VD in PDAC			
Authors	Year of Publication	Type of Study	Results/Conclusions
Zugmaier G. et al. [38]	1996	VD analog EB1089 and retinoids all-trans- and 9-cis-retinoic acid on cell lines	Combination of VD analogs and all-trans retinoic acid inhibited the growth of PDACcell lines
Pettersson F. et al. [39]	2000	Impact of: 9-cis-retinoic acid (9cRA) and VD analogs EB1089 and CB1093 on PDAC lines	VD analogs—inhibit PDAC cell growth 9cRA—induce apoptosis VD analogs—mechanism unclear Combination of VD and 9cRA—antagonistic
Albrechtsson et al. [40]	2003	Expression of VDR in human PDAC In vitro effect of EB 1089 on cell lines	High concentrations of EB 1089 caused reduction in number of cells in cell lines
Ohlsson B et al. [41]	2004	Impact of fat-soluble vitamins analogs on growth of PDACcell lines	High concentrations of vit A and D analogs decreased the cell number in PDACcell lines No increased impact on combining the two vitamins
Mouratidis et al. [42]	2006	Analysis of regulation of proteins involved in apoptotic signaling pathways in PDAC cells	Inhibitory impact of EB1089 on 9-cis RA-induced apoptosis lies upstream of caspase activation and could be associated with reduction in p27Kip1 protein levels
Li et al. [3]	2019	Analysis of impact of VD concentrations on expression level of VDR and cell cycle-related proteins CDKN1A (p21) and CDK1 in pancreatic cells and Panc-1 PDAC cells	Upregulation of VDR, CDKN1A, and CDK1 by VD analog in normal pancreatic cells but not in the advanced cancer cell line
Gorchs L. et al. [41]	2020	Analysis of impact of calcipotriol on the activation of human pancreatic CAFs and T cells using 2- and 3-dimensional (2D, 3D) cell culture models	Reduction in tumor-supportive activity of CAFs, and T cell effector functions by VD analog
Moz et al. [5]	2020	Analysis of role of calcipotriol in counterbalancing PDAC-induced and SMAD4-associated intracellular calcium [Ca^2+^]_i_ alterations and cytokines release	Alterations induced by PDAC cells in the [Ca^2+^]_i_ of immune cells can be partially reverted by calcipotriol treatment, which promotes inflammation and antagonizes PBMCs apoptosis
Cheng et al. [44]	2021	Human PDAC cells were treated with TNF-α in the presence or absence of 13-cis RA and VD	Suppression of TNF-α mediated cell invasion and potential PDAC prevention

VD, vitamin D; PDAC, pancreatic ductal adenocarcinoma; PBMCs, peripheral blood mononuclear cells; VDR, Vitamin D receptor; CAFs, cancer-associated fibroblasts; TNF-α, Tumor Necrosis Factor–alpha.

**Table 3:** A summary of the most important clinical studies on the association between sunlight exposure and pancreatic cancer.

Studies on Association Between VD Intake and PDAC			
Authors	Year of Publication	Type of Study	Results/Conclusions
Mohr et al. [45]	2010	Epidemiological study	Higher PDAC incidence rates in regions with lower UVB irradiance
Kato et al. [46]	1985	Epidemiological study	Higher mortality due to PDAC in northern Japan and in Scandinavian and other northern European countries
Kinoshita et al. [31]	2007	Epidemiological study	Low solar radiation and low temperature might be associated with a higher PDAC risk
Boscoe et al. [29]	2006	Epidemiological study	Inverse relationship between UVB irradiance and PDAC incidence and mortality
Garland et al. [47]	2016	Epidemiological study	Inverse association of UVB irradiance with PDAC incidence
Eryilmaz et al. [48]	2015	Retrospective study (139 PC patients)	Better OS in PC patients living in regions with more sunlight exposure

VD, vitamin D; PDAC, pancreatic ductal adenocarcinoma; UVB, ultraviolet B; OS, overall survival.

**Table 4:** A summary of the most important clinical studies on the association between intake of vitamin D and pancreatic cancer.

Studies on Association Between VD Intake and PDAC			
Authors	Year of Publication	Type of Study	Results/Conclusions
Liu et al. [49]	2018	Meta-analysis (25 studies, 1,213,821 patients)	Potential reduction in PC incidence by VD
Genkinger et al. [50]	2014	Meta-analysis (14 studies, 2212 patients)	No significant association between milk food and VD intake and PC risk
Skinner et al. [8]	2006	Prospective multicenter study (365 PC patients)	Higher VD intake related to a decreased PDAC risk
Waterhouse et al. [51]	2016	Multicenter study including 9 case–control studies (2963 PC, and 8527 controls)	Association between dietary VD consumption and higher PC risk

VD, vitamin D; PDAC, pancreatic ductal adenocarcinoma; UVB, ultraviolet B; OS, overall survival.

**Table 5:** A summary of the most important clinical studies on the association between vitamin D status and pancreatic cancer risk.

Studies on Association Between VD Status and PDAC Risk			
Authors	Year of Publication	Type of Study	Results/Conclusions
Wolpin et al. [63]	2012	Case–control study (451 PC patients, 1167 controls)	Higher plasma VD concentrations associated with lower PC risk
Yuan et al. [13]	2016	Prospective cohort study (493 PC patients)	Longer OS in PC patients with sufficient plasma 25(OH)D levels
Cho et al. [52]	2013	Retrospective study (178 PC patients)	VD concentration < 20 ng/mL related to poor prognosis in patients with stage III and IV PDAC
Liu et al. [53]	2013	Meta-analysis (9 studies, 1,206,011 patients)	No significant association between VD serum concentration and PC risk
Zhang et al. [54]	2017	Meta-analysis (12 studies)	No significant association between VD intake and PC risk
Stolzenberg-Solomon R.Z. et al. [9]	2006	Prospective case–control study (200 PC patients, 400 controls)	Higher VD concentrations related to a 3-fold increased PC risk
van Duijnhoven F.J.B. et al. [57]	2018	Multicenter case–control study (738 PC patients, 738 controls)	No significant association between increasing VD concentrations and higher PC risk
McGovern et al. [58]	2016	Retrospective study (1222 PC patients)	No significant association between serum 25(OH)D concentration and OS/TTP in PC patients
Stolzenberg-Solomon et al. [59]	2010	Multicenter case–control study (952 PC patients, 1333 controls)	High VD concentration (≥100 nmol/L) associated with 2-fold increased PC risk
Van Loon et al. [10]	2014	Prospective cohort study (256 PC patients)	VD deficiency more frequent in patients with new diagnosis of advanced PC
Rassmussen et al. [61]	2021	Prospective cohort study (1267 patients)	VD deficiency related to increased inflammatory biomarkers in all PC stages Positive association between VD concentrations and OS in the resected stage I and II patients, but not in advanced PC patients
Li et al. [62]	2023	Meta-analysis (2369 PC patients)	Positive association between OS, but not PFS, in advanced PC patients
Shen at al. [60]	2024	Meta-analysis (529,917 PC patients)	No association between higher VD concentrations and PC incidence Positive association between higher VD concentrations and OS in PC patients

VD, vitamin D; PDAC, pancreatic ductal adenocarcinoma; UVB, ultraviolet B; OS, overall survival; PFS, progression-free survival.

**Table 6:** A summary of the most important clinical studies on the genetic analysis of vitamin D in pancreatic cancer.

Studies on Genetic Analysis of VD in PDAC			
Authors	Year of Publication	Type of Study	Results/Conclusions
Anderson et al. [64]	2013	Population-based case–control study (628 PC patients, 1193 controls)	Potential associations between polymorphisms in VD-related pathway genes and PDAC risk
Piper et al. [65]	2015	Multicenter case–control study (295 PC patients, 590 controls)	No significant association between DBP concentration and PC risk Significantly higher PC risk in patients in highest VD group

VD, vitamin D; PDAC, pancreatic ductal adenocarcinoma; UVB, ultraviolet B; OS, overall survival.

**Table 7:** A summary of the most important studies on the supplementation of vitamin D with concurrent established chemotherapy in pancreatic cancer.

Studies on Combination of VD and Established Chemotherapy in PDAC			
Authors	Year of Publication	Type of Study	Results/Conclusions
Yu et al. [68]	2010	Human pancreatic cancer model study Calcitriol and gemcitabine	Calcitriol used with gemcitabine promotes caspase-dependent apoptosis, causing increased anti-tumor activity compared to either agent alone
Sherman et al. [25]	2014	Murine model study Calcipotriol and gemcitabine	Calcipotriol reduces markers of inflammation and fibrosis in pancreatitis and human tumor stroma
Mukai et al. [69]	2018	Retrospective study (86 PC patients following resection with/without CRT) VD and CRT	VD inactivates CAFs and improves efficacy of CRT in PDAC
Kang et al. [70]	2021	Design model study VDR modulators based on phenyl-pyrrolyl pentane and gemcitabine	VDR modulators combined with gemcitabine remodel PDAC TME and may promote anti-cancer effect of gemcitabine

VD, vitamin D; PDAC, pancreatic ductal adenocarcinoma; CRT, chemoradiotherapy; CAFs, cancer-associated fibroblast; TME, tumor microenvironment.

## Data Availability

The original contributions presented in this study are included in the article/Supplementary Material. Further inquiries can be directed to the corresponding author.

## Supplementary Materials

The following supporting information can be downloaded at: https://www.mdpi.com/article/10.3390/nu18050837/s1, Table S1. Integrated synthesis of discussed studies. Table S2. Limitations of existing evidence. Table S3. Future perspectives.
