# Supplementary material for: Impact of Vitamin D Status on Pancreatic Cancer Risk and Outcomes
Source: Nutrients. 2026 Mar 5;18(5):837. doi: 10.3390/nu18050837 (PMC12987229; doi:10.3390/nu18050837)
Supplement: Supplementary file 1 [file nutrients-18-00837-s001.zip › nutrients-4084562-supplementary.pdf]

MDPI

nutrients

Review

Impact of Vitamin D Status on Pancreatic Cancer Risk and Outcomes

Beata Jabłońska * and Sławomir Mrowiec

Department of Digestive Tract Surgery, Medical University of Silesia, Medyków 14, Katowice 40-752, Poland; mrowasm@poczta.onet.pl
* Correspondence: bjablonska@poczta.onet.pl

Supplementary Materials

Table S1. Integrated synthesis of discussed studies.

Table S2. Limitations of existing evidence.

Table S3. Future perspectives.

Table S1. Integrated synthesis of discussed studies.

Integrated Synthesis of Discussed Studies	
**Type of Studies**	**Results/Conclusions**
**Preclinical studies**	uniformly support the anti-tumorigenic actions of VD and VD analogs, showing inhibition of cancer cell proliferation, induction of apoptosis, modulation of stromal fibroblasts, and enhancement of chemotherapy efficacy.
**Ecological and observational studies**	linking sunlight/UVB exposure with PDAC incidence frequently indicate an inverse association, though they suffer from substantial confounding and ecological fallacies.
**Dietary intake studies**	reveal inconsistent results, with some showing protective effects and others demonstrating neutral or even adverse associations.
**Serum VD concentration studies**	are the most contradictory: some show improved PDAC outcomes with adequate VD, while others suggest increased risk with high VD levels.
**Genetic studies**	reveal that polymorphisms in VD-related genes may modulate PDAC susceptibility, underscoring the importance of individual metabolic differences.
**Collectively,**	the evidence points toward a complex, context-dependent relationship between VD and PDAC, likely influenced by genetic background, comorbidities, environmental factors, and the multifactorial nature of carcinogenesis.

VD, vitamin D; PDAC, pancreatic ductal adenocarcinoma.

Table S2. Limitations of existing evidence.

Limitations of Existing Evidence	
**Limitation**	**Description**
**Lack of randomized controlled trials (RCTs)**	Most human studies are observational, making causal inference impossible.
**Heterogeneity of methodologies**	Differences in study design, geographic regions, assessment of sunlight exposure, dietary intake, supplementation, and assays for serum 25(OH)D contribute to inconsistent findings.
**Confounding factors**	Obesity, nutritional status, dietary patterns, comorbidities, medications, outdoor physical activity, geographic localization, and skin phototype often are not fully controlled.
**Reverse causation**	Low VD levels may be a marker—not a cause—of systemic inflammation, cachexia, or underlying disease burden.
**Variation in VD metabolism**	Differences in liver/kidney function, vitamin D binding protein, polymorphisms in VD pathway genes, and use of VD analogs complicate interpretation.
**Inadequate adjustment in survival studies**	Important prognostic factors such as CA19-9 levels, tumor stage, surgical margins, and molecular markers are often not considered.
**Variability in dose and form of supplementation**	Studies rarely distinguish between cholecalciferol, calcifediol, or synthetic analogs, limiting comparability.
VD, vitamin D; CA19-9, carbohydrate antigen.	

Table S3. Future perspectives.

Future Perspectives	
**Field of perspective**	**Description**
**Well-designed RCTs**	Conduct **well-designed RCTs** to determine whether VD supplementation alters PDAC risk or affects treatment outcomes.
**Measurement techniques**	Standardize **measurement techniques** for serum 25(OH)D and define optimal thresholds for cancer prevention and treatment.
**VD source**	Differentiate between **VD obtained from sunlight, diet, supplementation, and analog use**, considering their distinct metabolic pathways.
**Genomic profiling**	Integrate **genomic profiling** to identify subgroups who may benefit from targeted VD interventions.
**Variation in VD metabolism**	Differences in liver/kidney function, vitamin D binding protein, polymorphisms in VD pathway genes, and use of VD analogs complicate interpretation.
**VD analogs exploration**	Explore **VD analogs** that retain anti-tumor functions without inducing hypercalcemia.
**VD analogs combination**	Investigate combination approaches using **VD analogs with immunotherapy, chemotherapy, and stroma-modulating strategies**.
**VD in early versus advanced PDAC**	Examine **the role of VD in early versus advanced PDAC**, as emerging evidence suggests stage-dependent effects.
**Longitudinal impact of VD supplementation**	Evaluate the **longitudinal impact** of VD supplementation on inflammatory status, metabolic health, and immune modulation.
**Personalized approaches**	Although preclinical studies provide strong biological rationale for VD as a potential adjunct in PDAC management, current clinical evidence remains insufficient to justify broad public health recommendations. Personalized approaches integrating VD status, genetic profile, comorbidities, and treatment modalities may be necessary to clarify the true role of VD in PDAC prevention and therapy.
VD, vitamin D; PDAC, pancreatic ductal adenocarcinoma.	
